# Research Progress in the Preparation of Transition Metal Sulfide Materials and Their Supercapacitor Performance

**DOI:** 10.3390/mi15070849

**Published:** 2024-06-29

**Authors:** Jin Yan, Jiancheng Lu, Yuxuan Sheng, Yin Sun, Dapeng Zhang

**Affiliations:** 1 Naval Architecture and Shipping College, Guangdong Ocean University, Zhanjiang 524088, China; 2 Guangdong Provincial Key Laboratory of Intelligent Equipment for South China Sea Marine Ranching, Guangdong Ocean University, Zhanjiang 524088, China; ysun@gdou.edu.cn; 3 College of Ocean Engineering and Energy, Guangdong Ocean University, Zhanjiang 524088, China; 4 School of Mechanical Engineering, Guangdong Ocean University, Zhanjiang 524088, China

**Keywords:** supercapacitor, electrode material, transition metal sulfide, energy density, power density

## Abstract

Transition metal sulfides are widely used in supercapacitor electrode materials and exhibit excellent performance because of their rich variety, low price, and high theoretical specific capacity. At present, the main methods to prepare transition metal sulfides include the hydrothermal method and the electrochemical method. In order to further improve their electrochemical performance, two aspects can be addressed. Firstly, by controllable synthesis of nanomaterials, porous structures and large surface areas can be achieved, thereby improving ion transport efficiency. Secondly, by combining transition metal sulfides with other energy storage materials, such as carbon materials and metal oxides, the synergy between different materials can be fully utilized. However, future research still needs to address some challenges. In order to guide further in-depth research, it is necessary to combine the current research-derived knowledge and propose a direction for future development of transition metal sulfide electrode materials.

## 1. Introduction

**Figure 1:**
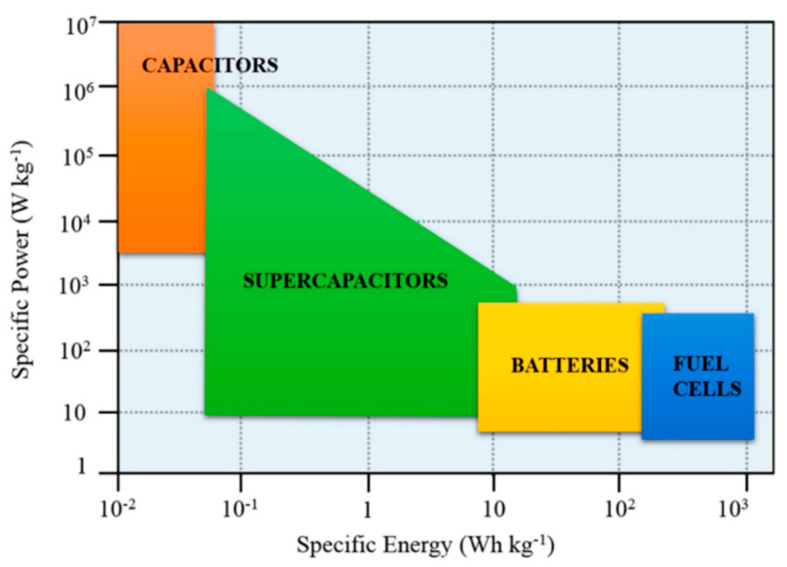
Ragone scheme for energy storage devices [2].

The global energy landscape is changing and experiencing several obstacles right now. The global energy pattern and techniques of energy usage are challenged by climate change, which is a result of greenhouse gas emissions on a worldwide scale. In addition, energy supply in some regions depends on specific countries or regions, something which will lead to supply shortages and price fluctuations. Furthermore, many nations have inadequate levels of energy waste and efficiency. Global demand for renewable energy has increased as a result, and nations have made strides in encouraging the growth of renewable energy to lessen reliance on fossil fuels and address the difficulties posed by climate change. Among them, the supercapacitor is a technology to improve energy efficiency and promote energy conservation (Figure 1). A supercapacitor is a type of energy storage device that has a high charge storage and release capacity. Supercapacitors provide several benefits over conventional batteries, including quick charge and discharge, extended longevity, high power density, low temperature characteristics, and environmental protection [1]. Its fast response capability and high-power output make it outstanding in scenarios that require frequent charge and discharge or high-power outputs. At the same time, long cycle life and environmental protection characteristics make it one of the important choices for sustainable development. Transition metal sulfides have the characteristics of metal compounds and excellent electrical conductivity. As a novel form of electrode material, it has lately shown considerable promise in the field of supercapacitors. This paper first introduces the concept of supercapacitors and then starts with the synthesis method of transition metal sulfides, leading to the importance of modification research. In view of the problems associated with transition metal sulfide electrode materials, such as easy agglomeration, small specific surface area, low conductivity, and poor cycle stability, the preparation of electrode materials with porous structure to promote electrolyte ion transport channels and large surface area for electrolyte ion storage is helpful to improve their electrochemical performance. A large number of studies have shown that the electrochemical properties of transition metal sulfide materials can be significantly improved by morphology control and compounding with other materials (Table 1).

## 2. Classification and Energy Storage Principle of Supercapacitors

Supercapacitors are usually classified into three categories based on the energy storage principle: asymmetric supercapacitors, pseudocapacitors, and electrochemical double-layer capacitors (EDLC).

### 2.1. Electrochemical Double-Layer Capacitor (EDLC)

**Figure 2:**
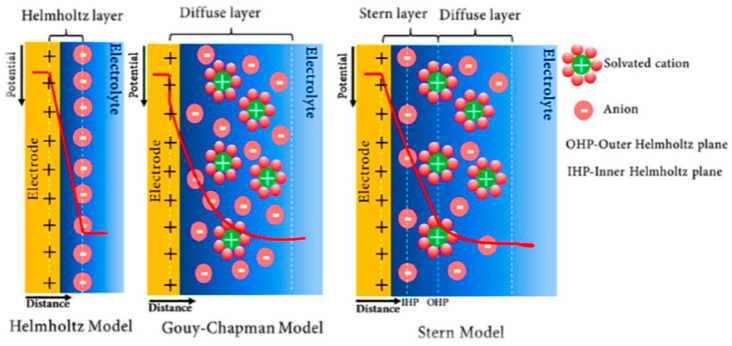
Schematics of the Helmholtz, Gouy–Chapman, and Gouy–Chapman–Stern EDLC models [9].

The way that ELDC works is explained by the principle that the charge stores energy in the double-layer region of the electrode surface. When the battery is charged, the positive and negative charges are separated and concentrated on the electrode surface to form a double layer composed of charges. During the charge process, the anions and cations in the electrolyte migrate to the anode and cathode, respectively, and their motions are opposite during the discharge phase [4]. The adsorption of electrolyte ions on the surface of a conductive electrode with a large specific surface area and porosity is the basis of the ELDC charge storage mechanism, which enables quick charging and discharging. This physical phenomenon enables ELDC to store energy efficiently [5]. The development of electric double-layer energy storage structure can be divided into three stages [6]. In 1953, Helmholtz first proposed the Helmholtz model. The model points out that there are opposite charges on both sides of the electrode interface [7] which are distributed in equal principle and divided by atomic detachment. This model can be equivalent to the traditional parallel plate capacitor model [8]. The Helmholtz model, however, fails to take the ion diffusion mechanism into account. On the basis of this, Gouy, Chapman, et al. further enhanced this model and presented the Gouy–Chapman model [9]. They introduced a new concept: the assumption that the distribution of ions (including cations and anions) near the electrode surface in the electrolyte solution is continuous, forming a tight layer, while the ions away from the electrode surface constitute a diffusion layer. Later, Stern proposed the ideas of the inner Helmholtz layer (IHP) and outer Helmholtz layer (OHP) and constructed the Stern model taking the volume factor of solution ions into consideration. This was done by combining the Helmholtz model with the Gouy–Chapman model. This model in Figure 2 unifies the idea of electric double-layer capacitance [10].

### 2.2. Pseudocapacitors

**Figure 3:**
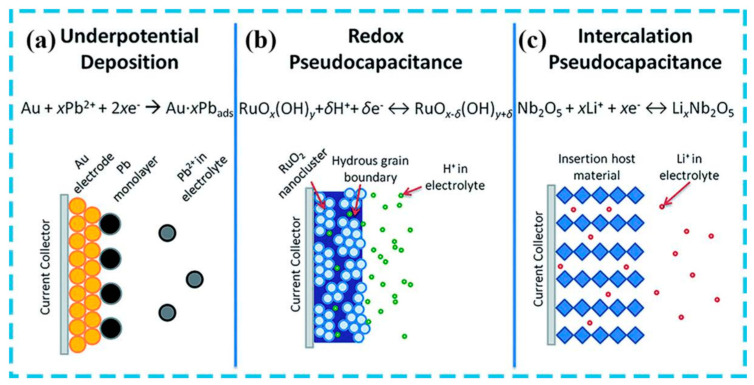
Schematic of EDLC models: Helmholtz, Gouy–Chapman, and Gouy–Chapman–Stern, respectively [13].

Pseudocapacitors store charges by chemical adsorption, desorption, or redox reactions and have characteristics similar to electrochemical capacitors. Similar to EDLC, the electrolyte ions build on the electrode surface throughout the charging process, and the adsorbed ions react oxidatively with the positive electrode’s surface molecules. The electrons travel in accordance with the reversible storage of ions during the discharge process [10]. In order to attain a larger energy storage density and, thus, an increased energy storage capacity, pseudocapacitors often employ materials with high specific surface area and electrochemical activity. They can be distinguished from EDLC by their pseudocapacitance storage method [11]. Pseudocapacitors employ a reversible redox reaction mechanism in conjunction with electrochemical adsorption and desorption to store charge, whereas EDLC uses physical adsorption of electrolyte ions. A faster discharge process is achieved by allowing ions to be adsorbed close to the material surface by Faraday charge transfer, which is made practicable by the ensuing surface redox activity. Compared with EDLC-based supercapacitors, pseudocapacitors have higher energy density and capacitance values (Figure 3). The structural integrity of the electrode material is preserved by ions tunneling into it during the Faraday transfer process [12].

### 2.3. Hybrid Supercapacitors

**Figure 4:**
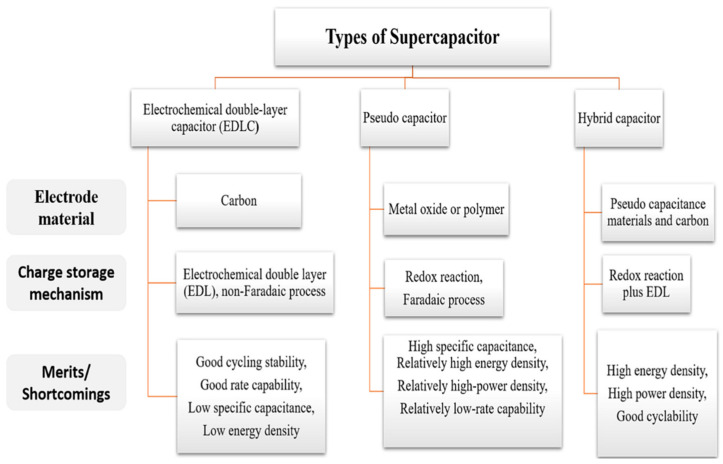
EDLC, pseudocapacitor, and hybrid capacitor comparison [15].

A hybrid supercapacitor is a variant of supercapacitor. Different energy storage mechanisms are included in the electrode materials utilized in the positive and negative electrodes [14]. This design can achieve higher energy density and better performance. Hybrid supercapacitors can achieve high energy density and high power density by choosing material combinations with various charge storage processes (Figure 4).

## 3. Supercapacitor Electrode Material

The electrode material selection principle of supercapacitors is conductivity, controllable porosity, high thermal stability, low cost, stability, and large effective surface area [16]. Appropriate electrode material selection is crucial, since the performance of these materials greatly influences the performance of supercapacitors. Carbon-based compounds and transition metal oxides are examples of common electrode materials. Pseudocapacitor supercapacitors can achieve larger specific capacities by using materials such as transition metal oxides, whereas EDLC typically uses carbon materials.

The cathode materials used in supercapacitors have a significant impact on their overall performance. The energy storage capacity of the device may be greatly increased by using cathode materials with a strong redox activity, excellent heat stability, good cycle stability, and good conductivity [17]. Supercapacitors often use metal oxides, carbon-based compounds, etc. as their cathode materials. One of the most popular types of electrode materials is carbon-based material, which has the benefits of high electronic conductivity, low cost, superior corrosion resistance, and cycle stability. The electric double layer created by the charge between the electrode and the electrolyte has a large specific surface area and is conducive to the adsorption and storage of electrolyte ions and is essential for the energy storage mechanism of the carbon-based electrode. As a new electrode material, transition metal sulfides have broad application prospects in the field of electrochemical energy storage. Compared with metal oxides, they have more redox active sites, better conductivity, and specific surface area, causing it to have higher specific capacity and better electrochemical performance. In addition, the weaker characteristic of an M–S bond compared to an M–O bond is beneficial to the occurrence of an electrochemical reaction. Multi-metal sulfides often exhibit better performance due to the change of valence state of multiple metals and the synergistic reaction between components. Therefore, transition metal sulfides are expected to become one of the important representatives of high-performance electrochemical energy storage materials in the future. Transition metal sulfides are widely used in electrochemical energy storage. However, metal sulfides face volume expansion and structural collapse in energy storage, leading to poor long-cycle stability and rate performance of the device. These shortcomings affect the practical application of metal sulfides in supercapacitors. Metal sulfides with different structures and morphologies have been synthesized through different preparation and improvement methods to improve their cycle and rate performance and promote the practical application of metal sulfides in supercapacitors [18].

Furthermore, the specific capacity and performance of carbon-based materials is also influenced by a specific surface area, pore size, and surface functionalization [19]. As a cathode material for supercapacitors, metal oxides have many advantages, such as low resistance and high specific capacitance. Transition metal oxides have drawn a lot of interest lately because of their superior chemical stability, greater specific capacitance, and higher energy density than carbon-based materials. Transition metal sulfides are very suitable for energy storage applications because of their outstanding thermal stability, high redox activity, and good electrical conductivity.

### 3.1. Synthesis Method of Transition Metal Sulfide Electrode Materials

The electrode materials of transition metal sulfides can be synthesized using the hydrothermal method or the electrochemical method. The morphology, particle size, specific surface area and electrochemical properties of the materials synthesized by different methods will change accordingly.

#### 3.1.1. Hydrothermal Method

**Figure 5:**
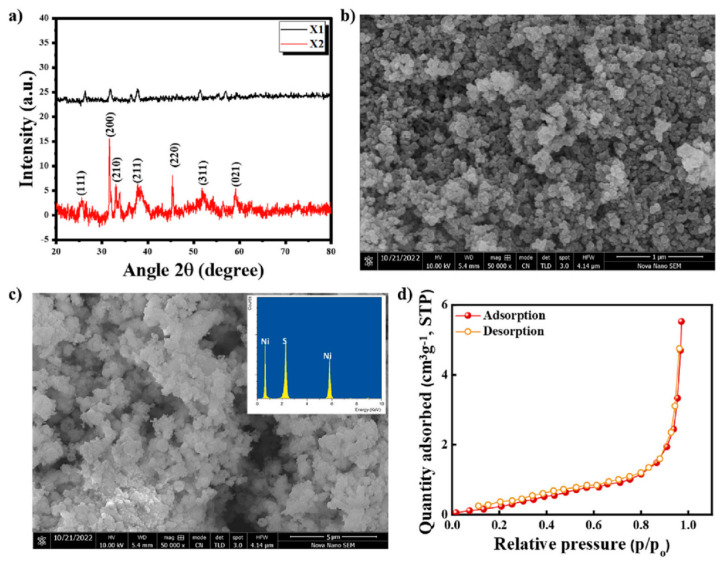
(**a**) Structure of the X1 and X2 samples as determined by the XRD spectrum. (**b**,**c**) Surface morphology as determined by the SEM at various magnifications; the inset shows the elemental analysis of NiS as determined by EDX. (**d**) BET of NiS [21].

The hydrothermal method is a simple and effective method for the synthesis of metal oxide materials. It uses hydrothermal or solvothermal synthesis routes to perform chemical reactions in a closed system and controls the morphology and properties of the product by adjusting parameters such as temperature, dissolved salt amount, and reaction time [20]. Iqbal et al. dissolved 0.045 M NiCl2·6H2O and 0.045 M Na_2_S·9H_2_O in 25 mL DI water, respectively, and stirred the solution in a magnetic stirrer for 30 min after mixing. After that, the mixture was placed in an autoclave and heated for a whole day in an oven. The NiS material was then recovered after the solution had been repeatedly cleaned with acetone, methanol, and deionized water (Figure 5). It was then dried for six hours at 65 °C in an oven to produce a crystalline powder of NiS materials [21].

**Figure 6:**
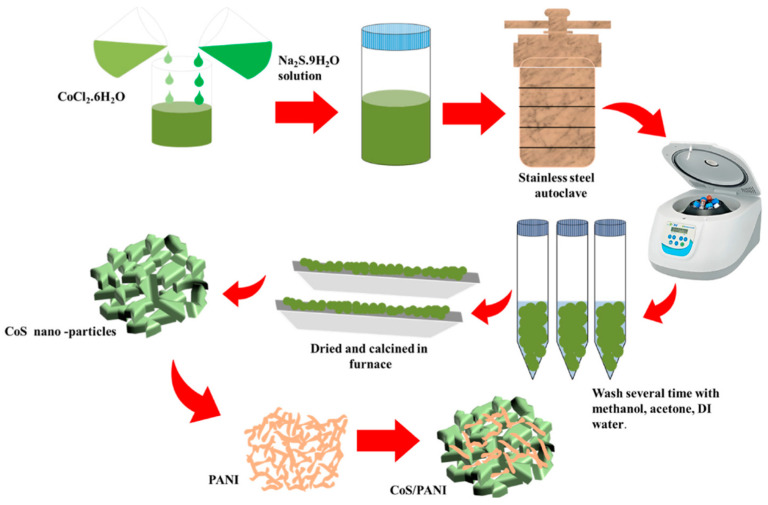
Schematic illustration of the CoS/PANI based on hydrothermal analysis [24].

Iqbal et al. (Figure 6) dissolved CoCl_2_·6H_2_O and Na_2_S·9H_2_O in deionized water to form a solution, then mixed and heated the solution in a stainless steel autoclave for 12 h. CoS was obtained by centrifuging the solution and annealing the precipitate at 80 °C [22]. The synthesized cobalt sulfide/polyaniline composites have excellent electrochemical properties. Cyclic voltammetry studies have shown that, at a scan rate of 3 mV/s, the composite material has a specific capacity of up to 490.4 C/g [23]. Additionally, it has been discovered that, by calculating the cyclic charge–discharge curve, the cobalt sulfide/polyaniline composite has a longer discharge time and a larger specific capacity, reaching 331.8 C/g, at a current density of 2 A/g. According to these findings, the composite material performs exceptionally well electrochemically in terms of cycle parameters [24].

**Figure 7:**
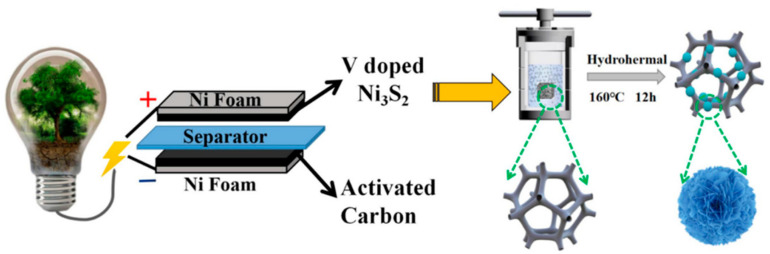
Schematic representation of the fabrication process of a VNS electrode [29].

Wang et al. used a two-step hydrothermal method to synthesize NCS. Firstly, nickel salt and cobalt salt containing different anionic groups were selected as raw materials to prepare NCS by a two-step hydrothermal method (Figure 7). The nickel–cobalt bimetallic sulfides synthesized from different anionic raw materials have different morphologies, among which NCS–C with perfect urchin-like morphology exhibits an excellent electrochemical performance. Its morphology has abundant redox reaction active sites, and the specific capacitance can reach 1112 F/g at a current density of 6 A/g [25].

The nickel foam was sliced into 1 × 1 cm^2^ by Yan et al. and the oxide layer and grease were removed from the surface by ultrasonically cleaning it for 30 min in acetone and 3 M hydrochloric acid [26]. It was then extensively cleaned with ethanol and deionized water before being dried for 12 h at 60 °C in an oven. In the synthesis process, anhydrous ethanol, thioacetamide, and ammonium metavanadate were mixed and added to the nickel foam. For a period of 12 h, the reaction was conducted in an autoclave set to 160 °C [27]. Following the synthesis, the samples were dried for 12 h at 60 °C in an oven after being cleaned with ethanol and deionized water [28]. After undergoing 10,000 continuous GCD tests at a current density of 2 A/g, the hybrid supercapacitor demonstrated exceptional cycle stability and an 82.2% capacitance retention rate [29].

The water bath heating method can improve the electrochemical performance of supercapacitors by optimizing the material synthesis process. For example, manganese-doped nickel-sulfide–tin-sulfide/reduced graphene oxide composites have been successfully synthesized, exhibiting a high specific capacity and cycle stability in supercapacitor devices. In addition, the water bath heating method can also be used to prepare negative electrode materials, such as binder-free manganese sulfide/reduced graphene oxide composites, to improve the performance of supercapacitor devices.

#### 3.1.2. Electrochemical Method

**Figure 8:**
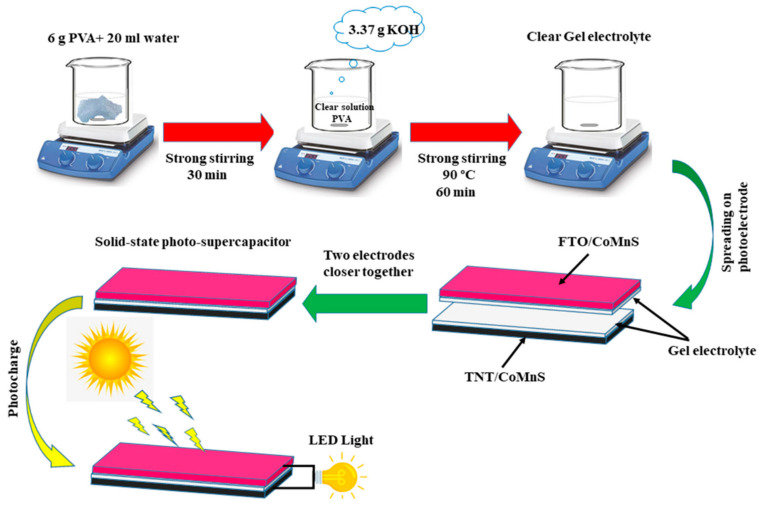
A schematic depiction of the method used to assemble an asymmetric supercapacitor cell (ASC) device [33].

The electrochemical encapsulation process uses the ions in the electrolyte to deposit on the electrode surface, forming a solid film or coating (Figure 8). During the electrochemical deposition process, a voltage or current is applied to the electrode surface to activate the redox reaction, causing charged ions to precipitate and deposit to produce the necessary material layer. Momeni et al. effectively constructed Co, Mn, and Co–Mn–S-based porous photoresponsive heterostructures on the surface of anodized TiO_2_ nanotubes by electrochemical deposition [30]. Researchers electrodeposited CoS, MnS, and CoMnS onto the surface of anodized TiO_2_ nanotubes [31]. At a current density of 0.70 mA/cm^2^, the electrochemical capacitance values of TNTs, CoS/TNTs, MnS/TNTs, and the best CoMnS/TNTs under dark circumstances are 20 mF/cm^2^, 31.3 mF/cm^2^, 22.5 mF/cm^2^, and 42.2 mF/cm^2^, respectively [32,33].

**Figure 9:**
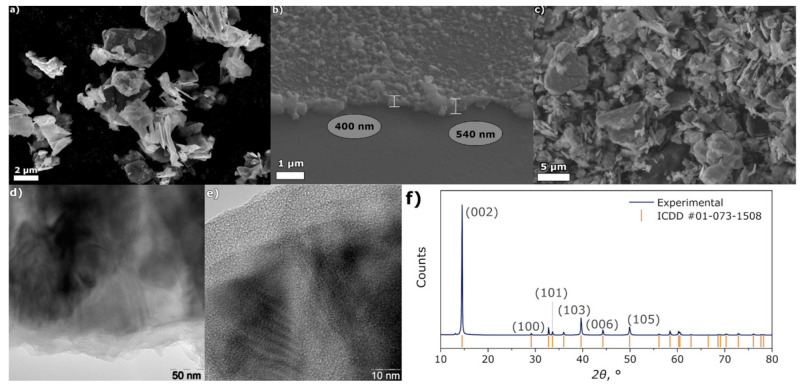
SEM images of the following: (**a**) pure MoS_2_ powder, (**b**) the surface of the PEDOT/MoS_2_ composite film, (**c**) the edge of the film at a 45° angle displaying the polymer thickness, (**d**,**e**) TEM images of MoS_2_ platelets implanted into PEDOT at different magnifications, (**f**) the pristine MoS_2_ powder’s XRD spectrum [37].

Alexey I. Volkov et al. synthesized PEDOT/MoS_2_ composites using two methods: in situ oxidation EDOT polymerization and electrochemical polymerization [34]. In the electrochemical synthesis process, the commercially available MoS_2_ was dispersed in the EDOT acetonitrile solution and the composite material was deposited by capturing MoS_2_ in the electropolymerized PEDOT matrix. A glassy carbon electrode is positioned upwards in an inverted electrochemical cell during the synthesis process and either a dynamic potential or constant current deposition mode is used to develop a composite film [35]. Before the synthesis, MoS_2_ particles are dispersed in solution and ultrasonically treated to prevent agglomeration (Figure 9). The electrodeposition process involves the deposition of MoS_2_ particles on the electrode surface [36]. Subsequently, the growth of the conductive polymer layer and the combination of the precipitate form a composite film. The PEDOT/MoS_2_ composite synthesized by electrochemical deposition method has been shown to exhibit good electrochemical performance in 1 mol/dm^3^ LiClO_4_ aqueous solution. The PEDOT/MoS_2_ composite has also been shown to maintain an initial capacitance of 87% after 100 cycles and 71% after 350 cycles [37].

The electrochemical deposition method has been widely used in the field of material research, especially in the preparation of materials with excellent electrochemical properties. High-performance electrode materials can be prepared by electrochemical deposition, such as Mn-doped nickel–cobalt sulfide (Mn0.5-NCS) which exhibits an excellent performance in supercapacitors and catalysts.

#### 3.1.3. Comparison of Hydrothermal Method and Electrochemical Method

The electrochemical method and hydrothermal method are two commonly used methods for synthesizing and preparing materials [38]. They have their own unique properties and range of applications and are frequently employed in the field of material research. Therefore, in the preparation and research of materials, appropriate methods can be selected for experiments and applications according to specific needs [39].

### 3.2. Study on the Modification of Transition Metal Sulfide Electrode Materials

The utilization of metal sulfide electrodes is limited by their rapid agglomeration, which results in low conductivity, small specific surface area, and poor cycle stability [40]. The development of a porous structure will aid in the transfer of electrolyte ions, and an increase in surface area will improve electrode material performance as well as electrolyte ion storage [41]. Studies have shown that the electrochemical properties of metal sulfide electrode materials are improved by morphology control and compounding [42].

#### 3.2.1. Morphology Control of Transition Metal Sulfide Electrode Materials

The shape of transition metal sulfides as electrode materials has a significant impact on their electrochemical performance [43]. An electrode material’s ability to store energy is influenced by its specific surface area, electrical conductivity, and ion transport characteristics, all of which could be regulated by morphological control [44]. It is pointed out that the specific capacity of supercapacitors can be significantly enhanced by adjusting the pore size and distribution, so as to improve their performance. In addition, recent studies have shown that maximizing the electrode surface area is essential for achieving high specific capacitance of supercapacitors. This is because the electrode material with high surface area can provide more active sites, something which is helpful for the adsorption of charge and the diffusion of electrolyte ions, thereby reducing the equivalent series resistance and increasing the power density. Therefore, an optimal design of electrode morphology and pore size is crucial to improve the performance of electrode materials. The pore size has a significant effect on the specific capacity of the electrode, and the ideal pore size range is usually between 1 nm and 2 nm. In this range, high ion accessible surface area and effective charge storage can be provided. In addition, recent studies have also shown that reducing the crystallographic diameter of the pore relative to the ion can enhance the capacitance. This is because the highly distorted solvent cladding/pores can bring ions closer to the electrode surface, thereby increasing the capacitance. Composite electrode materials having a core–shell structure can be developed to increase the stability and performance of electrode materials [45,46].

**Figure 10:**
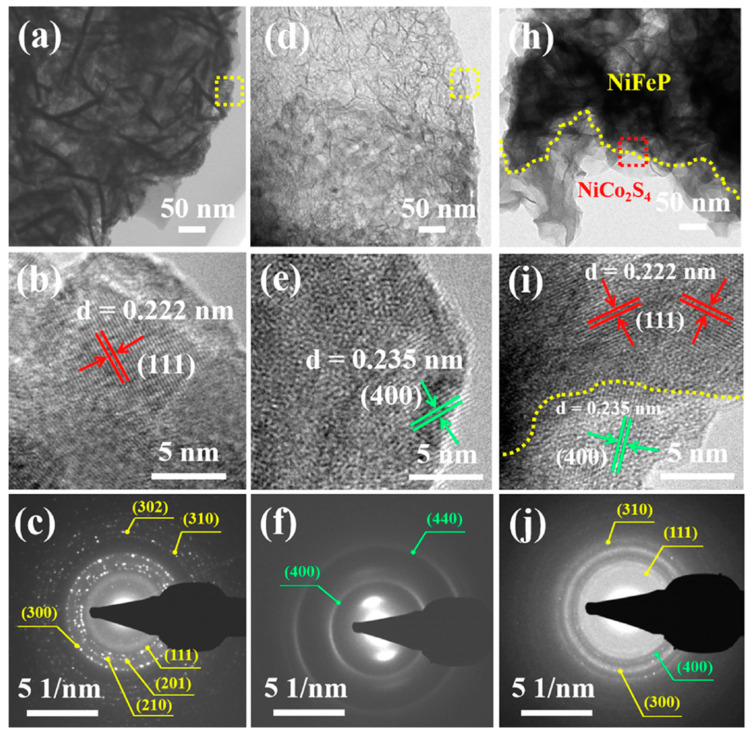
TEM images of (**a**,**b**) NiFeP nanosheets, (**d**,**e**) NiCo2S4 nanosheets, and (**h**,**i**) NiFeP@NiCo2S4 hy-brid nanosheets at low and high magnifications, and their corresponding SEAD patterns of (**c**) NiFeP, (**f**) NiCo2S4, and (**j**) NiFeP@NiCo2S4 [51].

Liu et al. successfully grew NiCo_2_S_4_ nanosheets on the surface of NiFeP/CC to form a NiFeP@NiCo_2_S_4_ mixed nanosheet structure [47]. These hybrid nanosheets have a core–shell structure, with the core composed of NiFeP nanosheets and the shell composed of NiCo_2_S_4_ nanosheets [48]. Based on observation, a highly porous structure composed of evenly coated NiFeP and NiCo_2_S_4_ nanosheets could be observed on the surface of CC (Figure 10). The electrode material’s volume expansion and shrinkage during repeated electrochemical reactions were lessened by this hierarchical structure, which also helps expose a large number of active sites [49,50]. The NiFeP@NiCo_2_S_4_/CC hybrid electrode continued to exhibit good cycle stability after 5000 cycles at a current density of 5 A/g, as per the experimental results [51].

**Figure 11:**
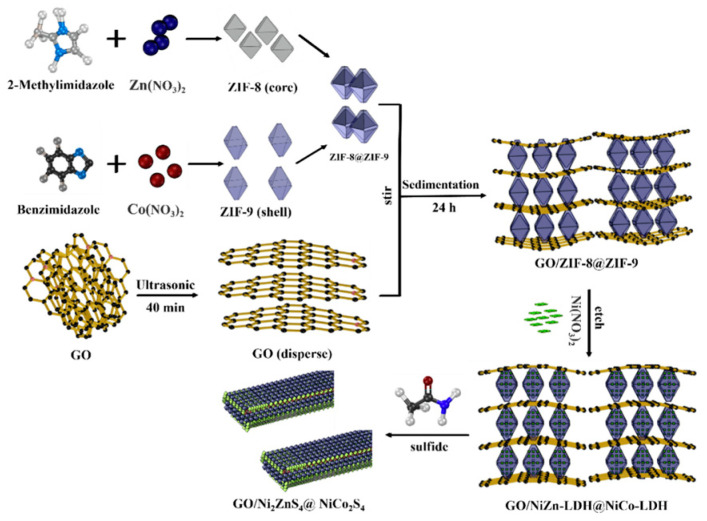
Schematic of GO/Ni_2_ZnS_4_@NiCo_2_S_4_ and GO/NiZn-LDH@NiCo_2_-LDH [60].

Cui et al. (Figure 11) synthesized ZIF-9 using the solvothermal method, then added ZIF-9 to the reaction system of 2-MI and Zn(NO_3_)_2_·6H_2_O and added GO for in situ compounding to achieve GO/ZIF-8@ZIF-9. Then, through Ni(NO_3_)_2_·6H_2_O etching [52,53], Ni^2+^ reacted with OH^−^, partially replacing Zn^2+^ and Co^2+^, and with numerous free Zn ions and Co ions and partial Ni ions on ZIF-8 and ZIF-9 to form NiZn-LDH@NiCo-LDH. Finally, GO/Ni_2_ZnS_4_@NiCo_2_S_4_, with white fungus-like core–shell structure, was obtained by hydrothermal vulcanization [54].

**Figure 12:**
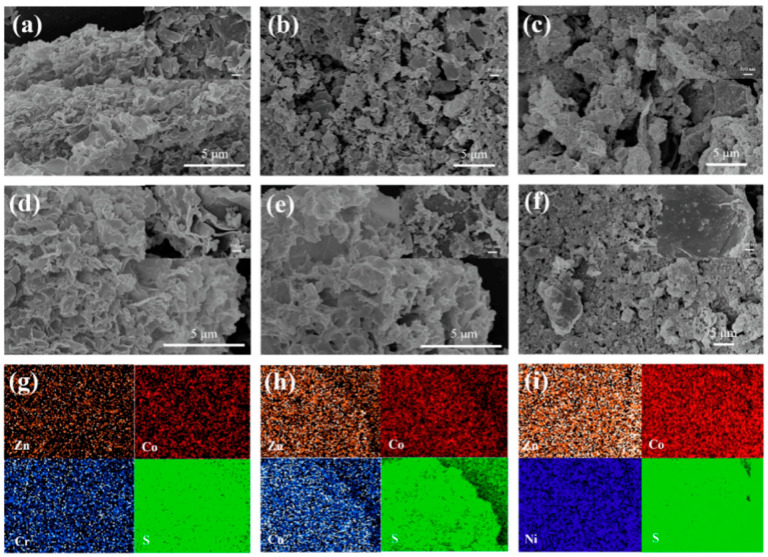
(**a**) GO/CrZn-LDH@CrCo-LDH, (**b**) GO/CuZn-LDH@CuCo-LDH, (**c**) GO/NiZn-LDH@NiCo-LDH, (**d**) GO/Cr_2_ZnS_4_@Cr_2_CoS_4_, (**e**) GO/Cu_3_ZnS_4_@CuCo_2_S_4_, (**f**) GO/Ni_2_ZnS4@NiCo_2_S_4,_ and elemental mapping images of (**g**) GO/Cr_2_ZnS4@Cr_2_CoS_4_, (**h**) GO/Cu_3_ZnS_4_@CuCo_2_S_4_, and (**i**) GO/Ni_2_ZnS_4_@ NiCo_2_S_4_ [60].

The experimental findings demonstrate that high temperature carbonization and hydrothermal vulcanization were successful in producing GO/Ni_2_ZnS_4_@NiCo_2_S_4_ composites with numerous active sites and strong conductivity [55]. In addition to enhancing the activity of the aforementioned composites, the inclusion of graphene oxide generates a loose, porous, layered structure that boosts the electrochemical performance and electron transport capacity [56]. With a core–shell structure, Ni_2_ZnS_4_@NiCo_2_S_4_ generates more active sites and ion transport channels [57]. The GO/Ni_2_ZnS_4_@NiCo_2_S_4_ electrode shown in Figure 12 has been shown to have a good rate performance and be able to produce a reversible capacitance of 1902.5 F/g after 5000 cycles at a current density of 1 A/g [58,59]. At a power density of 750 W/kg, the ASC made up of the BC cathode and GO/Ni_2_ZnS_4_@NiCo_2_S_4_ anode has been shown to have a maximum energy density of 168.7 Wh/kg [60].

Li et al. synthesized Co-MOF by dissolving Co(NO_3_)_2_·6H_2_O and 2-methylimidazole in 100 mL methanol and stirring for 10 min. They then rapidly added the 2-methylimidazole solution to the metal salt solution and stirred for 30 min to obtain a uniformly purple solution. Lastly, they collected, washed, and dried the mixture [61]. The preparation of Co–C requires carbonization and annealing of Co-MOF in nitrogen for 3 h, with the calcification temperature adjusted as needed to change the morphology of the sample [62]. Lastly, to prepare Co-C@NiCoB, a solution of Ni(NO_3_)_2_·6H_2_O (0.1 M), Co(NO_3_)_2_·6H_2_O (0.1 M), and 1 mL of NaBH_4_ must be mixed. A total of 10 mg, 20 mg, 30 mg, 40 mg, and 50 mg of Co–C should be added to it, with the solution stirred in an ice water bath for half an hour. Then, another mixed solution should be added and stirred for another half an hour or until no bubbles form. The Co-C@NiCoB electrode is then gathered, cleaned, and allowed to dry. The Co-C@NiCoB electrode has been shown to have a mass load of 1.5 mg/cm^2^ [63,64].

**Figure 13:**
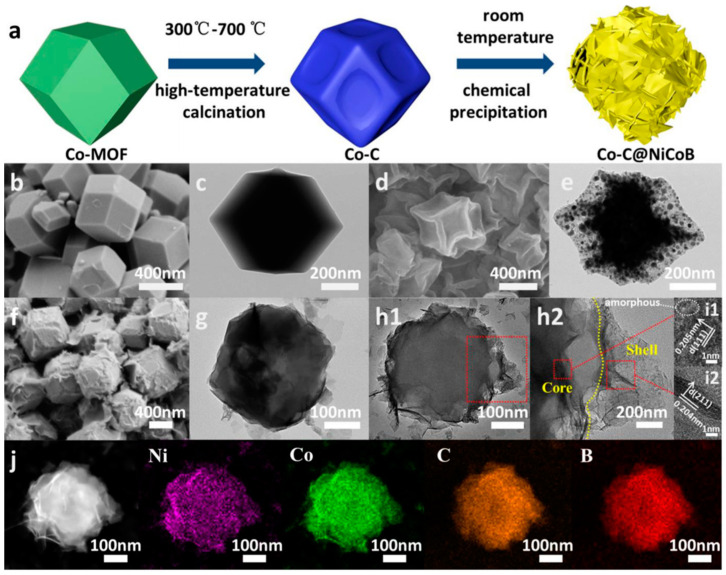
Co–C@NiCoB’s preparation process is covered in (**a**); Co–MOF’s SEM and TEM are covered in (**b**,**c**); Co-C’s SEM and TEM are covered in (**d**,**e**); Co–C is covered in (**f**,**g**) with SEM and TEM of Co–C@NiCoB; Co–C@NiCoB’s TEM and magnified TEM are covered in (**h1**,**h2**); HRTEM of Co-C@NiCoB (**i1**,**i2**); Co–C@NiCoB’s element mapping is shown (**j**) [69].

The ASC device has been shown to retain its original specific capacitance of 92.1% after 10,000 charge–discharge cycles at a current density of 1 A/g, demonstrating good cycle stability. Figure 13a,b shows the nanostructure.In the initial 10 cycles of charge and discharge, the gadget has been shown to exhibit remarkable stability and reversibility [65,66]. The structural integrity of the Co-C@NiCoB electrode has been demonstrated by TEM pictures, which display the electrode’s core–shell structure after 10,000 cycles [67,68]. Furthermore, the resistance of the ASC device is measured before and after the cycle. Results reveal that the slope of the EIS graph remained steady at low frequencies, indicating that the device exhibited great electrochemical stability [69].

Studies have shown that high specific capacity and excellent cycle stability of supercapacitors can be achieved by constructing electrode materials with a core–shell structure. This core–shell structure opens up a new performance optimization approach in the field of supercapacitors, which helps to improve the conductivity and charge transfer efficiency of materials, thereby enhancing energy storage density and cycle life. The introduction of a core–shell structure can significantly improve the performance of electrode materials, providing a new possibility for the future development of electrochemical energy storage technology.

The charge–discharge rate and performance response speed of electrode materials are enhanced by nanosheets’ shorter electron transport and ion diffusion routes, both of which are made possible by their nanoscale properties [70]. Additionally, nanosheets are highly structured and have a significantly larger surface area, both of which are advantageous for increasing the electrode material’s specific surface area and its area of contact with the electrolyte, in turn, promoting the electrochemical reaction [71,72]. Nanosheets could significantly decrease the volume expansion and shrinkage of electrode materials during charging and discharging, as well as enhance cycle stability and durability due to their high flexibility and plasticity [73]. Li et al. firstly prepared a CMS integrated cathode using a two-step hydrothermal method, and then immersed the CMS integrated cathode in an NaBH4 solution to form Vs-CMS. Nanosheets with different surface morphologies can be successfully prepared by adjusting the concentration of NaBH4 [74].

**Figure 14:**
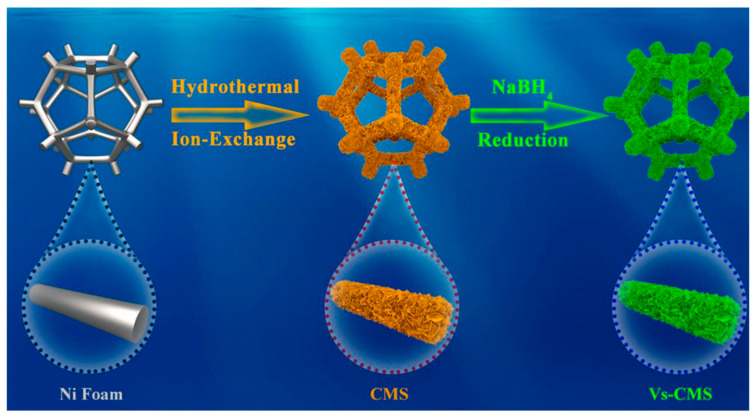
Diagrammatic representation of the Vs-CMS integrated cathode synthesis process [78].

**Figure 15:**
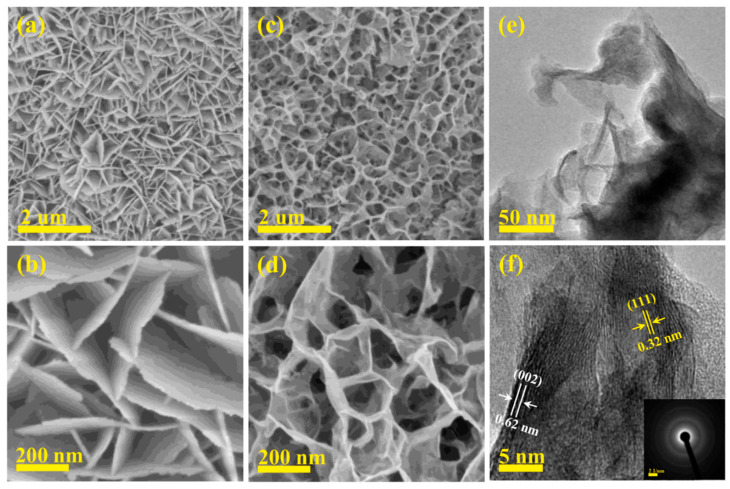
(**a**) low- and (**b**) high-resolution FESEM images for CMS integrated cathode; (**c**) low- and (**d**) high-resolution FESEM images for Vs-CMS-0.6 integrated cathode; (**e**) low-resolution TEM and (**f**) HRTEM images for Vs-CMS-0.6 integrated cathode (inset shows the corresponding SAED spec-trum [78].

With the increase in NaBH4 concentration, the surface microstructure of the nanosheets will change, the surface will become rougher, and more defects will appear [75]. This is because NaBH4 has a potent reduction impact that can lead to a large number of defects on CMS, altering the surface morphology [76]. Experimental results have shown that even low concentrations of NaBH_4_ can change the surface microstructure, and, with the increase in NaBH_4_ concentration, the distortion degree of Vs-CMS relative to CMS has also been shown to increase [77]. Compared to CMO (81.9%) and CMS (92.6%), Vs-CMS-0.6 might keep 96.7% of its original capacity after 10,000 cycles, according to the testing data. Furthermore, during the cycle stability test, the η values of CMO, CMS, and Vs-CMS-0.6 have been shown to be almost 100%, suggesting that they have excellent electrochemical reaction reversibility [78]. Figure 14 shows the preparation process and Figure 15a,b shows the nanostructures. The Vs-CMS integrated cathode has a high area capacity, good rate performance, and excellent cycle stability. However, at high current densities, the charge–discharge time may be shortened, which may be due to the sluggish Faraday redox kinetics of battery-type electrode materials, resulting in insufficient Faraday redox reaction at high current densities.

**Figure 16:**
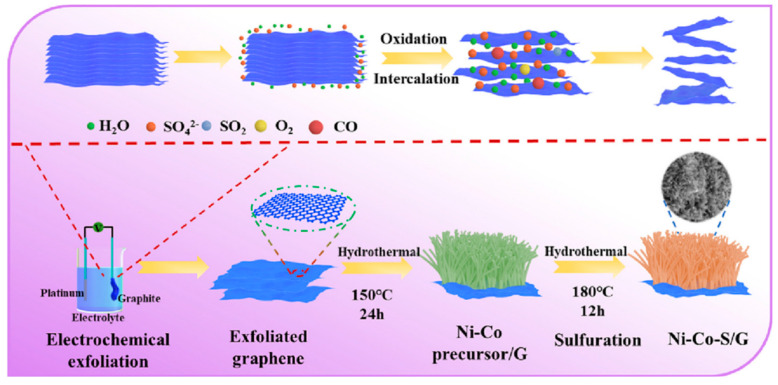
A schematic for Ni–Co–S/G preparation [83].

**Figure 17:**
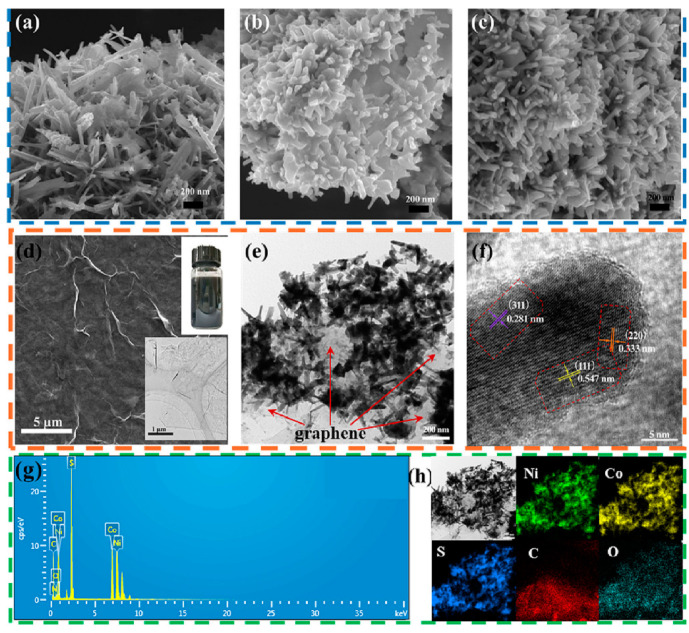
Samples with typical SEM pictures include (**a**) Ni–Co–S, (**b**) B–Ni–Co–S/G, (**c**) Ni–Co–S/G, (**d**) SEM and TEM images of exfoliated graphene, (**e**,**f**) TEM and HR-TEM images of Ni–Co–S/G, (**g**) EDS spectra, and (**h**) corresponding elemental mappings of Ni–Co–S/G [83].

Jin et al. first prepared graphene by electrochemical exfoliation [79]. Then, the nickel–cobalt precursor was mixed with graphene dispersion and the Ni–Co precursor/G was prepared under high temperature conditions. Finally, the sulfurized urea was added to the mixture to prepare Ni–Co–S/G under high temperature conditions [80]. Experimental results have shown that, when nickel–cobalt sulfide nanorods are formed on graphene, a well-designed electrode material could offer a large number of active sites for the Faraday reaction as well as a more efficient electron/ion transport channel during charge and discharge [81]. High specific capacity of 1579.68 F/g at 1 A/g, outstanding rate performance of 1240 F/g at 20 A/g, and great cycle stability of 91.5% after 5000 cycles at 5 A/g are all displayed by the Ni–Co–S/G electrode. Furthermore, with a power density of 1125 W/kg, the asymmetric supercapacitor built using commercial activated carbon and Ni–Co–S/G as positive and negative electrodes has shown an energy density of 75.3 Wh/kg [82]. Figure 16 shows the fabrication process. These superior electrochemical characteristics suggest that the Ni–Co–S/G that was produced is a potentially useful pseudocapacitive material for supercapacitors [83]. The a-b of Figure 17 shows the nanostructure.

New nanomaterials can help solve the challenges of supercapacitors by providing more active sites to promote Faraday redox reactions and accelerate electron and ion transport. For example, the introduction of sulfur vacancy-enhanced cobalt molybdenum sulfide nanosheets (Vs-CMS) can improve the energy density and cycle stability of supercapacitors. In addition, layered transition metal sulfide nanosheets (such as MnCo_2_S_4_) have also been proved to be an integrated cathode material to improve the electrochemical performance of supercapacitors. Nanomaterials for supercapacitors can include different structural forms such as carbon nanotubes, graphene, and activated carbon. As a one-dimensional structure, carbon nanotubes have high conductivity and a low cost and can provide good charge transport channels, so they have potential application prospects in supercapacitors. Graphene, as a two-dimensional structure, has excellent electrical conductivity and thermal properties which can improve the performance of supercapacitors, especially in composites with metal oxides which can increase specific capacitance and energy density. Although activated carbon has low conductivity, it is also one of the commonly used materials in supercapacitors as a three-dimensional structure with a high specific surface area and high specific capacitance. Therefore, the structure of nanomaterials has an important influence on the performance and energy density of supercapacitors. Different nanomaterials play different roles in supercapacitors. The application of nanocomposites can make up for the shortcomings of single materials and give full play to their respective advantages to improve electrode performance. The research and application of nanocomposites have opened up new ways and possibilities to improve the performance of electrodes. By combining different materials into nanocomposites, higher specific capacity and cycle stability can be achieved, thereby improving the overall performance of the electrode. The introduction of advanced materials with high specific surface area and conductivity, such as graphene, carbon nanotubes, etc., can effectively overcome the problem of insufficient interlayer spacing of metal sulfides and improve their charge–discharge kinetic performance. These advanced materials can increase the active sites and promote ion transport, thereby significantly improving the energy storage performance of metal sulfides. After forming a composite structure with metal sulfides, the synergistic effect between advanced materials and metal sulfides can improve the structural stability of electrode materials and improve cycle stability. In addition, the construction of a core–shell and other composite structures also helps give full play to the respective advantages of core materials and shell materials and further enhance the electrochemical performance. Therefore, the introduction of advanced materials helps improve the conductivity and electron transport capacity of metal sulfide electrodes, thereby increasing their power density and energy density. Table 2 shows the electrochemical properties of transition metal sulfides.

#### 3.2.2. Composite of Metal Sulfide Electrode Materials

**Figure 18:**
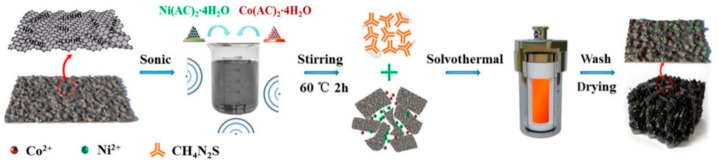
The typical synthesis of NiCo_2_S_4_@rGO [86].

To address the issues of inadequate conductivity, volume expansion, and poor cycle stability in metal sulfides, researchers are investigating a range of approaches, including surface coatings, nano structuring, and the use of conductive additives. Metal sulfides can be compounded with other materials to enhance their electrochemical performance, cycle life, and charge–discharge performance. Graphene is a highly conductive substance that, when paired with metal sulfides, may greatly increase the electrode’s overall conductivity, encourage electron transit, lower resistance, and enhance the electrode’s electrochemical performance [84]. Graphite provides good physical support and charge transport channels in supercapacitors. The transition metal sulfides have a large specific surface area and excellent electrochemical activity, both of which are expected to improve the capacitance and cycle stability. The combination of these materials can make full use of the conductivity of graphite and the energy storage properties of transition metal sulfides, thereby improving the performance of supercapacitors. Zhao et al. first brought the pH of a 70 mL graphite oxide solution down to 10. After that, they put the solution in an autoclave and heated it for six hours at 120 °C. Subsequently, N-doped graphene was freeze-dried and collected after being cleaned with deionized water. With one amp per gram, the GS-NCS-0.5 electrode has a high specific capacity of 2418 F/g. The asymmetric supercapacitor, when constructed into a device, has a power density of 411 W/kg and an energy density of 34.1 Wh/kg. As seen in Figure 18, the device capacitance has a respectable cycle stability, losing just 13.6% of its original value after 5000 cycles. The working potential window in the aqueous electrolyte was increased to 1.6 V [85,86].

**Figure 19:**
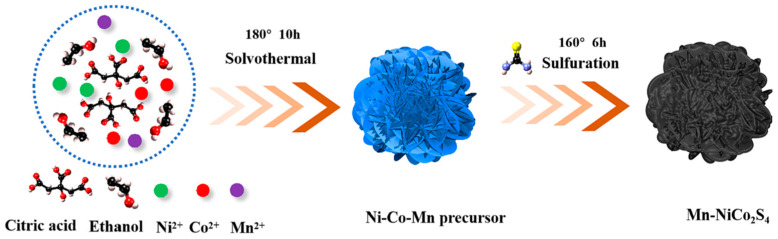
An illustration of the formation process for Mn-NCS [89].

By using metal compounding, one may extend the life of the battery, decrease capacity attenuation during charge and discharge, and enhance the cycle stability of metal sulfides. Xu et al. mixed 2 mmol Ni(NO_3_)_2_·6H_2_O, 4 mmol Co(NO_3_)_2_·6H_2_O, and 2 mmol citric acid (CA) and liquefied the resulting mixture into a 40 mL solution comprising 10 mL H_2_O and 30 mL C_2_H_6_O [87]. After a certain time of magnetic stirring, a uniform solution was formed. The mixture was then transferred to a 100 mL PTFE-lined stainless-steel autoclave and subjected to solvothermal treatment at 180 °C for 10 h. The treated precipitate was recovered by centrifugation and then washed with deionized H_2_O and C_2_H_6_O multiple times to eliminate surface impurities [88]. Then, it was dried in a vacuum oven at 70 °C for 12 h. After 7000 cycles at a high current density of 5 A/g, the capacity retention rate was 81% (Figure 19) and the columbic efficiency was close to 100% [89].

Song et al. firstly added nickel chloride, cobalt chloride, and thioacetamide to ethylene glycol and then added different concentrations of graphene oxide, stirring for 1 h and ultrasonicating for 2 h. Then, the optimized CoNiS-10GO electrode was fabricated by a simple one-step hydrothermal method, which showed large specific capacitance and good cycle stability [90].

**Figure 20:**
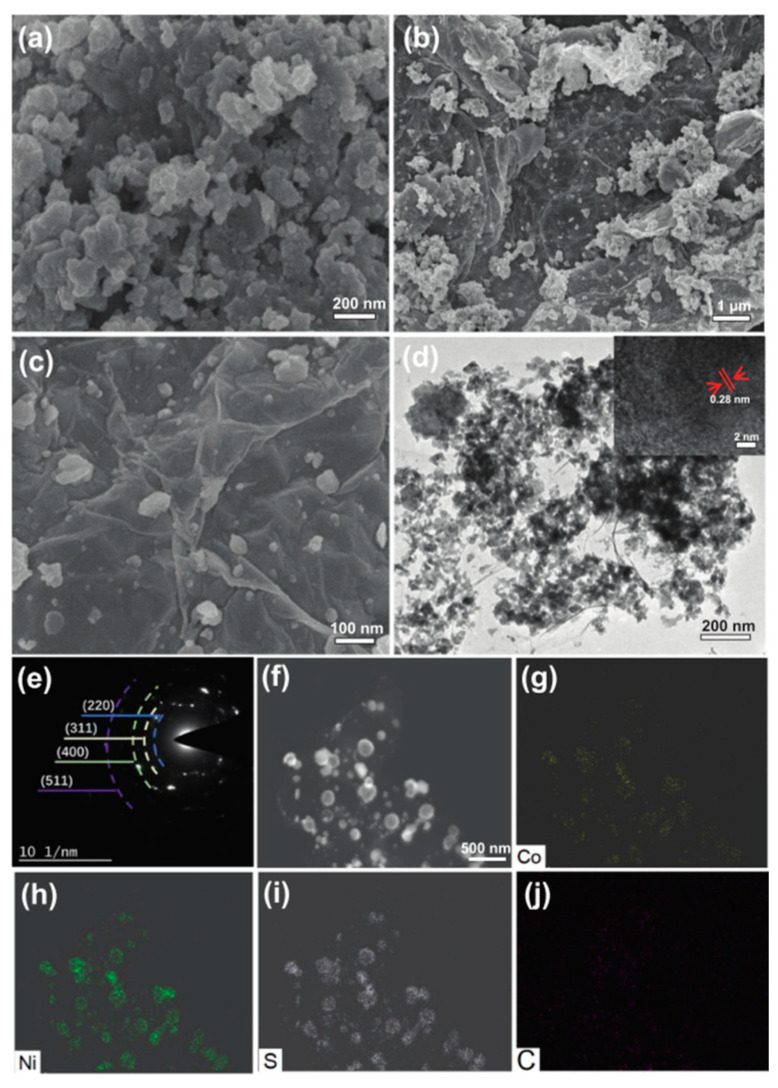
SEM image of (**a**) CoNiS, (**b**) CoNiS-10GO. TEM image of (**c**) CoNiS and (**d**) CoNiS-10GO. (**e**–**j**) SAED pattern and mapping images of CoNiS-10GO [92].

The CoNiS-10GO electrode performs exceptionally well in supercapacitors. The electrode has a good rate performance with a specific capacitance of up to 746.7 C/g at 1 A/g and a retention rate of 69.3% at 20 A/g. Furthermore, in the PVA/KOH solution electrolyte, the asymmetric supercapacitor with porous carbon as the negative electrode and CoNiS-10GO as the positive electrode has shown high cycle stability and a substantial energy density. The findings shown in Figure 20 offer a valuable framework for the conceptualization and creation of novel electrochemical energy storage apparatuses [91,92].

In the process of synthesizing cathode materials, Paul et al. first mixed the aqueous solution comprising NiCl2·6H2O and SnCl_2_·2H_2_O under magnetic stirring for about 30 min. The solution was then supplemented with about 0.4 M of thioacetamide aqueous solution and approximately 0.4 M of MnCl_2_ aqueous solution. Following a gentle dropwise addition of these solutions to an aqueous dispersion of graphene oxide at a concentration of 2 mg/mL, the combined solution was put in a 100 mL hydrothermal reactor and allowed to react for 24 h at 120 °C [93,94]. The finished item was collected for characterization, vacuum-dried, and thoroughly cleaned. The aqueous solution comprising 0.1 M Mn(NO_3_)_2_·4H_2_O and 0.1 M TAA was first combined under magnetic stirring for 30 min in order to create the synthetic anode material. After that, the produced solution was put into a 100 cc autoclave reactor filled with bare nickel foam and it was kept there for around 24 h at a temperature of around 120 °C [95,96]. After washing, the product was gathered and given the name MS. In order to treat MSG, nickel foam was first impregnated, then covered with an aqueous dispersion containing 2 mg/mL of graphene oxide [97].

**Figure 21:**
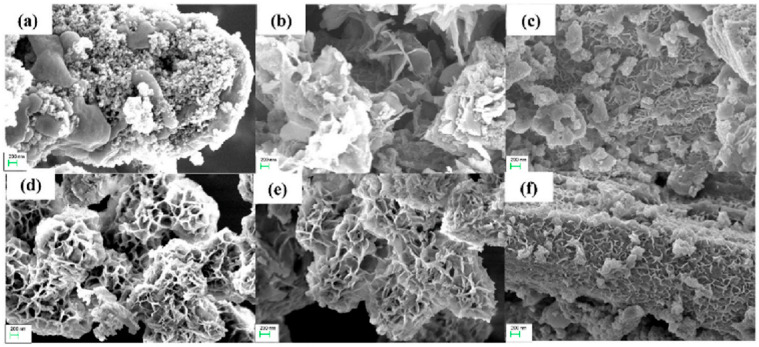
FE-SEM images of (**a**) NS1, (**b**) NS2, (**c**) NS3, (**d**) MNS1, (**e**) MNS2, and (**f**) MNS3 [100].

According to the experimental results, Mn-doped NiS-SnS/rGO composite (MNS) was successfully synthesized by a one-step hydrothermal method as a cathode material. In this composite, manganese is used as a dopant to improve its electrochemical activity, as it can produce more active sites, due to the composite’s poor electronegativity and range of oxidation levels [98]. For anode materials, binder-free manganese sulfide/reduced graphene oxide composites (MSG) are grown in situ on the surface of nickel foam. In experiments, MNS has been shown to work in the range of 0–0.6 V at a current density of 2 A/g in 1 M Na_2_SO_4_ electrolyte, providing a specific capacitance of about 810 F/g. In the same electrolyte, MSG provides a specific capacitance of about 390 F/g in the range of −0.85–0 V at a current density of 2 A/g [99]. At a power density of 1086 W/kg, the supercapacitor device built with MNS as the cathode material and MSG as the anode material has shown a high energy density of around 50.37 Wh/kg. Furthermore, Figure 21 illustrates that, even after 10,000 GCD cycles, the supercapacitor device retains a specific capacitance of almost 85% [100].

The combination of metal oxides and carbon materials helps improve the performance of supercapacitors because metal oxides are the main energy storage materials, while carbon materials provide physical support and charge transport channels. For example, researchers have synthesized a composite of copper oxide nanoribbons and carbon nanotubes with a specific capacitance of 150 F/g. The composites of manganese oxide and carbon nanotubes have obtained specific capacitances of 250 F/g and 184 F/g at scan rates of 10 mV/s and 100 mV/s, respectively. In addition, graphene and its composites with metal oxides have, thus far, shown good potential, because graphene has good electrical conductivity, thermal properties, and a large surface area, all of which can improve the performance of supercapacitors. The composites of metal oxide and graphene oxide have also shown high specific capacitance. For example, the specific capacitance of the composites of iron oxide and graphene oxide have been shown to reach 504 F/g. The design and synthesis of manganese-doped nickel-sulfide–tin-sulfide/reduced graphene oxide composites as cathode materials and manganese sulfide/reduced graphene oxide composites as anode materials help achieve high energy density and cycle stability. These composites are designed to improve the energy density of supercapacitor devices and achieve high energy density and cycle stability by optimizing the capacitance of positive and negative electrode materials. Therefore, metal composite materials provide an effective way to improve the energy density of supercapacitors. Metal and transition metal sulfide composites show potential application prospects in the field of supercapacitors. Transition metal sulfides are considered to be powerful candidates for electrode materials because of their high theoretical specific capacity and excellent electrical conductivity, both of which help improve energy storage density. At the same time, metals provide the function of physical support and charge transport channels, while transition metal sulfides bear the main responsibility of charge and energy storage. Through their combination, we can give full play to the advantages of the two and make up for their shortcomings. Studies have shown that metal and transition metal sulfide composites can significantly increase the specific capacity of capacitors and improve cycle stability, laying an important foundation for the future development of energy storage. Table 3 shows the electrochemical properties of transition metal sulfides.

## 4. Conclusions

Supercapacitors have emerged as one of the most significant electrochemical energy storage technologies due to their high-speed charge and discharge, extended life cycle, high power density, environmental protection, and extensive commercial application opportunities [101]. Transition metal sulfides are a class of functional materials that have many potential uses and research applications because of their unique structures and superior electrochemical, catalytic, and photoelectric qualities [102]. In supercapacitors, transition metal sulfides have superior electrochemical performance and cycle stability [103]. The conductivity of metal sulfides may be greatly increased, electron transit encouraged, and resistance decreased by compounding with other materials, such as graphene, a process which can enhance the electrode’s electrochemical performance [104]. The metal sulfide composite material exhibits exceptional cycle stability, rate performance, and specific capacity, suggesting possible uses in supercapacitors [105]. In addition, metal sulfide nanomaterials have shorter electron passage and ion diffusion paths [106], both of which are conducive to rapid electron transportation and ion diffusion, and improve the charge–discharge rate and enactment response speed of electrode materials. Future studies can investigate the raw materials, procedures, and structure–property connection of metal sulfides in greater detail. They can also identify strategies for achieving controlled synthesis of electrode materials [107]. Regulating the structure of metal sulfide nanoparticles is crucial to improve their electrochemical performance. By rationally designing the composition and morphology of metal sulfides and compounding with carbon-based materials, the performance of metal sulfides in electrochemical energy storage systems can be significantly enhanced. At present, a variety of colloidal synthesis methods have been established to prepare high-yield metal sulfide nanomaterials, but the structure growth mechanism of some metal sulfides (such as nanoboxes, nanospheres, nanotubes, etc.) is still not very clear. Therefore, in the future, it is necessary to further study these reaction mechanisms to find suitable methods to synthesize the desired structures. Surface modification has been shown to improve the performance of metal sulfides. Therefore, it is still an important research direction to develop simple synthesis methods to prepare surface functionalized metal sulfides with controllable size and morphology. In the future, it is necessary to understand the reaction mechanism of transition metal sulfides, because the reaction mechanism is crucial to improve the performance of transition metal sulfides in energy storage systems. First of all, the growth mechanism of transition metal sulfide nanoparticles with different structures (such as nanoboxes, nanospheres, nanotubes, etc.) is still unclear. In-depth study of these mechanisms will help find a suitable synthesis method to obtain the required structure and properties. In addition, transition metal sulfides are prone to structural damage during the electrochemical process, resulting in performance degradation. Therefore, understanding the reaction mechanism is helpful to design a reasonable surface modification strategy and improve its cycle stability. Finally, transition metal sulfides will form unstable lithium/sodium/potassium compounds in the battery, causing problems such as volume expansion. Studying its reaction mechanism will help solve these problems and improve the cycle performance of the battery.

Additionally, by utilizing the synergistic impact between the components, metal sulfides could be combined with other materials to enhance the electrochemical performance. In the design and preparation of electrode materials, materials characterized by a rich content, low price, and environmental friendliness are selected, and they may be applied to a wider range of fields. Although transition-metal-sulfide-based electrode materials have great potential in improving electrochemical performance, they also face many scientific challenges. Two of the main problems are energy density and poor conductivity: the energy density of supercapacitors is one of the main limits to their development. Therefore, there are two main strategies to improve the energy density of supercapacitors: adjusting the capacitance or adjusting the voltage. The capacitance is affected by the selected dielectric material, electrode surface area, and double-layer capacitance thickness. By increasing the specific surface area of the electrode, the capacitance of the device can be effectively improved. Another way to increase the energy density is to increase the voltage, because the energy density is proportional to the square of the voltage. Therefore, changing the electrolyte or manufacturing a hybrid supercapacitor can adjust the voltage. At present, the use of new electrolytes, such as organic electrolytes or ionic electrolytes, has become one of the effective methods to improve the energy density of supercapacitors. In addition, the electrochemical properties of supercapacitors can also be changed by adjusting electrode materials or electrolytes. Another problem is poor conductivity, which limits the performance as a positive electrode in hybrid supercapacitors. In addition, the complex synthesis process, difficult preparation methods, and high cost also restrict the large-scale application of industrial production. Potential ways to address these challenges include introducing conductive additives or adjusting the material structure to improve electrical conductivity, as well as developing lower-cost synthetic methods suitable for large-scale production. In addition, designing a more stable electrode material and controlling the volume expansion during the cycle will help extend the life of the material. Increasing the number of active sites of redox reaction and improving chemical stability are also key. Therefore, in order to ensure the sustainable development and wide applicability of hybrid supercapacitors, researchers should give priority to the use of environmentally friendly, low-cost, and resource-rich materials.

In the future, transition metal sulfide materials with different morphologies and specific surface areas should be synthesized, and nanomaterials with adjustable particle size should be synthesized by electrodeposition on conductive substrates to increase the number of active sites and improve electron transport, thereby improving electrode stability. At the same time, the construction of core–shell composite structures and the synergistic effect of core materials and shell materials can improve the structural stability and cycle stability of the electrode, thereby improving the stability of the electrode. In addition, the introduction of highly conductive materials such as graphene and carbon nanotubes to enhance the electron transport capacity of the electrode material can improve the energy storage performance and cycle stability of the electrode, thereby further improving the stability of the electrode. In addition, the introduction of two or more metal ions into transition metal sulfides to form binary or ternary metal sulfides can increase the electron concentration and conductivity, thereby improving the electrochemical performance and stability of the electrode. Finally, the synthesis of transition metal sulfide nanowire arrays by simple and environmentally friendly methods such as ion exchange can improve the specific capacity and cycle stability of the material, thereby further improving the stability of the electrode. These methods will bring new possibilities for the application of transition metal sulfide materials in electrochemical energy storage systems and promote their development in the future energy field.

## Figures and Tables

**Table 1:** An analysis comparing the key features of batteries, supercapacitors, and traditional capacitors [3].

Characteristic	Battery	Supercapacitor	Conventional Capacitor
Charging/ discharging time	1 to 10 h	Milliseconds to seconds	Picoseconds to milliseconds
Energy density	8 to 600 Wh/kg	1 to 10 Wh/kg	0.01 to 0.05 Wh/kg
Power density	0.005 to 0.4 Wh/kg	1 to 120 Wh/kg	0.25 to 10,000 Wh/kg
Weight	1 to >10 kg	0.001 to 0.230 kg	1 to 10 kg
Operating voltage	1.25 to 4.2 V	6 to 800 V	2.3 to 2.75 V
Life	150 to 1500 cycles	50,000+ h unlimited cycles	>100,000 cycles

**Table 2:** Study on electrochemical properties of transition metal sulfides.

Synthetic Material	Specific Capacitance (Fg^−1^)	Current Density (Ag^−1^)	Retention (%)	Cycle (No.)	Energy Density (Whkg^−1^)	Power Density (Wkg^−1^)	Ref.
NiFeP@NiCo_2_S_4_	874.4	5	91.2	5000	32.1	18,034.2	[51]
GO/Ni_2_ZnS_4_@ NiCo_2_S_4_	218	1	62.5	5000	168.68	750	[60]
Co-C@NiCoB	211	1	92.1	10,000	66.03	763.35	[69]
Vs-CMS	395.1	1	96.7	10,000	73.2	252.2	[78]
Ni–Co–S/G	1579.68	1	91.5	5000	75.3	1125	[83]

**Table 3:** Study on electrochemical properties of transition metal sulfides.

Synthetic Material	Specific Capacitance (Fg^−1^)	Current Density (Ag^−1^)	Retention (%)	Cycle (No.)	Energy Density (Whkg^−1^)	Power Density (Wkg^−1^)	Ref.
GS-NCS-0.5//N-rGO	2418	1	86.4	5000	34.1	411	[86]
Mn0.5-NCS	156	1	81	7000	55.4	797	[89]
CoNiS-10GO	746.7	1	80.9	5000	37.3	——	[92]
MNS_2_	810	2	85	10,000	50.37	1086	[100]
